# Tremor suppression following treatment with MRgFUS: skull density ratio consistency and degree of posterior dentatorubrothalamic tract lesioning predicts long-term clinical outcomes in essential tremor

**DOI:** 10.3389/fneur.2023.1129430

**Published:** 2023-04-25

**Authors:** Kain Kyle, Jerome Maller, Yael Barnett, Benjamin Jonker, Michael Barnett, Arkiev D’Souza, Fernando Calamante, Joel Maamary, James Peters, Chenyu Wang, Stephen Tisch

**Affiliations:** ^1^Brain and Mind Centre, The University of Sydney, Sydney, NSW, Australia; ^2^Sydney Neuroimaging Analysis Centre, Camperdown, NSW, Australia; ^3^GE Healthcare, Melbourne, VIC, Australia; ^4^Department of Medical Imaging, and Neurology, St Vincent’s Hospital, Darlinghurst, NSW, Australia; ^5^Department of Neurology, St Vincent’s Hospital, Darlinghurst, NSW, Australia; ^6^Department of Neurosurgery, St Vincent’s Hospital, Darlinghurst, NSW, Australia; ^7^Royal Prince Alfred Institute of Academic Surgery, University of Sydney, Camperdown, NSW, Australia; ^8^Department of Neurology, Royal Prince Alfred Hospital, Camperdown, NSW, Australia; ^9^School of Biomedical Engineering, The University of Sydney, Sydney, NSW, Australia; ^10^Sydney Imaging, The University of Sydney, Sydney, NSW, Australia; ^11^School of Medicine, University of New South Wales, Sydney, NSW, Australia

**Keywords:** vim, dentatorubrothalamic tract, essential tremor, MRgFUS, tremor

## Abstract

**Objectives:**

Magnetic resonance-guided focussed ultrasound (MRgFUS) is an incisionless ablative procedure, widely used for treatment of Parkinsonian and Essential Tremor (ET). Enhanced understanding of the patient- and treatment-specific factors that influence sustained long-term tremor suppression could help clinicians achieve superior outcomes *via* improved patient screening and treatment strategy.

**Methods:**

We retrospectively analysed data from 31 subjects with ET, treated with MRgFUS at a single centre. Tremor severity was assessed with parts A, B and C of the Clinical Rating Scale for Tremor (CRST) as well as the combined CRST. Tremor in the dominant and non-dominant hand was assessed with Hand Tremor Scores (HTS), derived from the CRST. Pre- and post-treatment imaging data were analysed to determine ablation volume overlap with automated thalamic segmentations, and the dentatorubrothalamic tract (DRTT) and compared with percentage change in CRST and HTS following treatment.

**Results:**

Tremor symptoms were significantly reduced following treatment. Combined pre-treatment CRST (mean: 60.7 ± 17.3) and HTS (mean: 19.2 ± 5.7) improved by an average of 45.5 and 62.6%, respectively. Percentage change in CRST was found to be significantly negatively associated with age (β = −0.375, *p* = 0.015), and SDR standard deviation (SDR_SD_; β = −0.324, *p* = 0.006), and positively associated with ablation overlap with the posterior DRTT (β = 0.535, *p* < 0.001). Percentage HTS improvement in the dominant hand decreased significantly with older age (β = −0.576, *p* < 0.01).

**Conclusion:**

Our results suggest that increased lesioning of the posterior region of the DRTT could result in greater improvements in combined CRST and non-dominant hand HTS, and that subjects with lower SDR standard deviation tended to experience greater improvement in combined CRST.

## 1. Introduction

Tremor is the result of oscillatory neuronal activity in the brain, manifesting as a rhythmic contraction of muscles leading to involuntary movement in various parts of the body. While the generation, propagation and amplification of this neuronal activity are not completely understood, the involvement of particular regions and networks has been identified, and these regions have become targets for surgical treatments of tremor (1).

The ventral intermediate nucleus (Vim) of the thalamus is a critically component in tremor networks (2–4), acting as a junction between cerebellar and cortical pathways, receiving afferent projections from the contralateral deep cerebellar nuclei *via* the dentatorubrothalamic tract (DRTT) and connecting to the ipsilateral motor cortex. It has been shown that through surgical lesioning (5) or deep brain stimulation (DBS) (3, 6) of the Vim, tremor suppression can be achieved in patients with Essential Tremor (ET).

Magnetic Resonance-Guided Focused Ultrasound (MRgFUS) is an incisionless ablative procedure that in recent years has been applied to the treatment of tremor (7). MRgFUS uses the targeted delivery of ultrasonic waves to deliver focussed energy to tissue in the brain, causing thermal necrosis resulting in the destruction of tissue at the focal point of the ultrasound waves. Combining focussed ultrasound (FUS) with structural magnetic resonance imaging (MRI) and MR thermometry allows accurate placement of this focal point in the appropriate region for optimal tremor suppression.

The Vim is a common target for treatment of tremor with MRgFUS, and accurate localisation of the target is imperative for successful tremor suppression. Direct targeting of the Vim is complicated by the fact that the borders of the Vim are not visible on standard structural imaging using 3 T MRI. The traditionally accepted approach is to use indirect targeting, in which the Vim location is inferred by stereotactic landmarks; however, this approach does not fully account for individual variations in anatomy or the effects of global or regional brain atrophy (8, 9).

Neuroimaging based methods for improved targeting of the Vim is an ongoing area of research. Automated thalamic segmentation tools, such as THOMAS (10) and the segmentation algorithm developed by Iglesias et al. (11), available in the popular neuroimaging software suite FreeSurfer (12), generate segmentations of the individual thalamic nuclei, including the ventral posterior nuclei (VLp), the ventral portion of which includes the Vim (13). These segmentation algorithms may benefit clinicians by providing additional information on the otherwise indistinguishable boundaries between each thalamic nuclei.

More recently, diffusion weighted imaging (DWI) tractography has emerged as a useful technique in defining the connectivity of the thalamus to aid in target selection. Many of the findings from studies of DBS have demonstrated proximity to the DWI tractography generated DRTT and the correlation with tremor suppression (14–18), knowledge which has been translated to treatment with MRgFUS. In MRgFUS studies, the strongest evidence to date of DRTT involvement comes from several studies examining the microstructural changes along the DRTT and the relationship with clinical outcomes. Thaler et al. observed that fractional anisotropy (FA) changes at the ablation site and along the DRTT correlated with clinical improvement (19). Similarly, both Kapadia et al. (20) and Pineda-Pardo et al. (21) found that FA reduction along the DRTT was correlated with tremor improvement, and that these changes were correlated with the overlap between the DRTT and the ablated volume.

While tractography and thalamic segmentation based methods for treatment targeting are promising, there remains a lack of consensus around the optimal method and parameters for accurate target definition. In this study, we investigated the overlap between the volume of tissue ablated with FUS and both the FreeSurfer and THOMAS thalamic segmentations, as well as with the tractography derived DRTT, to examine any relationship with tremor suppression. Additionally, we apply a streamline clustering algorithm to conventially generated DRTT streamlines with the aim of probing the relationship between tremor suppression and ablation of streamlines within the DRTT. We also include patient- and treatment-specific parameters in the statistical analysis, to determine which characteristics are most predictive of improved clinical outcomes to further aid in patient screening and treatment strategy.

## 2. Materials and methods

### 2.1. Subjects

This study was a retrospective analysis of data collected from 31 ET patients undergoing MR-guided focused ultrasound thalamotomy, targeting the Vim, and in a subset of patients either the posterior subthalamic area (PSA) or ventralis oralis anterior (VOA) nucleus, for treatment of tremor at St Vincent’s Hospital Sydney (Australia) between March 2019 and February 2021. All patients met 2018 consensus classification criteria (22) for ET or ET-plus, herein considered collectively as ET. In accordance with 2018 consensus criteria, patients classified as ET-plus met criteria for ET but had additional features including subtle dystonia, rest tremor, mild gait ataxia or mild Parkinsonian features. Informed consent was obtained from all participating subjects, and the study was approved by the St Vincent’s Hospital Ethics Review Committee (ETH00670). Patient characteristics are summarised in Table 1.

**Table 1:** Patient characteristics.

Variable	All patients
	*N* = 31
**Sex, *n*(%)**	
Male	23 (74)
Female	8 (26)
Age, mean	75.72 ± 7.20
SD, years	
**Treatment target, *n*(%)**	
Vim	22 (71)
Vim and PSA	8 (26)
Vim, PSA, and VOA	1 (3)

### 2.2. MRgFUS procedure

All subjects were assessed by a neurologist prior to participation in the study. Preoperative CT and MR imaging was acquired for estimation of skull density ratio (SDR) and stereotactic planning. Ultrasonic lesioning was performed using the ExAblate Neuro system (InSightec, Tirat Carmel, Israel) 650 kHz, with a 1,024-element, phased array ultrasound transducer. Imaging was simultaneously performed on a 3 T MRI scanner (SIGNA Architect, General Electric, Milwaukee). During each sonication, MRI thermometry was acquired at 3 s intervals to monitor the peak and mean temperature of tissue at the target site.

The initial target coordinates for the Vim were chosen by a neurosurgeon using the conventional stereotactic location 25% of the AC-PC distance anterior to the PC, 14 mm lateral to the midline at the level of the intercommissural line and adjusted for individual patient anatomy including width of 3^rd^ ventricle, and proximity to visualised internal capsule. The target location was refined using a series of low temperature (40^°^C–45^°^C), non-destructive sonications, with MR thermometry feedback to confirm target accuracy. Upon confirmation of the target, the energy of sonication was gradually increased to achieve temperatures between 53°C–63°C, creating a permanent ablation. Following each high energy sonication, the subject was evaluated to monitor tremor suppression and any side effects. These high energy sonication’s were repeated until maximal achievable tremor suppression with at least 2 sonications in the permanent lesion temperature range applied to the target.

In 9 of the 31 participants (Table 1), the PSA was additionally targeted where intra-procedural tremor suppression was incomplete after adequate VIM lesioning. In these patients, additional lesioning in PSA conferred additional tremor suppression, including proximal upper limb tremor, resulting in a greater clinical benefit at the conclusion of treatment. The coordinates of PSA were similar to those used for PSA DBS, targeting the white matter equidistant between the medial border of the STN and lateral border of the red nucleus at its equator, corresponding to AC-PC coordinates of approximately x = 9.5 mm, y = −6.0 mm, z = −5.5 mm. The VOA was additionally targeted in one ET-plus patient with dystonic tremor, in whom tremor relief was incomplete after VIM lesioning alone. In this patient, the coordinates for VOA lesioning were 12 mm right of midline, 11 mm anterior to the posterior commissure and 1.5 mm above the commissural plane. Analysis of the effect of these secondary lesions was beyond the scope of this investigation.

### 2.3. Imaging protocol

MR imaging was acquired 1–7 days prior to treatment and again the day after treatment. Pre-treatment imaging was acquired on a 3 Tesla MRI scanner (SIGNA Architect, General Electric, Milwaukee), and post-treatment imaging was acquired on a 3 T Philips Ingenia (Philips Inc., Amsterdam, The Netherlands).

The pre-treatment imaging protocol included a sagittal 3D IR-FSPGR T1-WI (TI: 450 ms, TR: 8 ms, TE:3.24 ms, Flip Angle: 10^°^, FOV: 256 mm, acquisition matrix: 256 × 256, slice thickness: 1.2 mm, slices: 146). Additionally, three different DWI protocols were acquired; Protocol 1 (*N* = 9): b = 0, 1,000 s/mm^2^, directions = 64, TR: 9,100 ms, TE: 90 ms, Flip Angle: 90^°^, FOV: 230 mm, acquisition matrix: 128 × 128, slice thickness: 1.8 mm, slices: 72. Protocol 2 (*N* = 15): b = 0, 700, 1,000, 2,800 s/mm^2^, directions = 140, TR: 6250 ms, TE: 106 ms, Flip Angle: 90^°^, FOV: 230 mm, acquisition matrix: 128 × 128, slice thickness: 1.8 mm, slices: 72. Protocol 3 (*N* = 7): b = 0, 700, 1,000, 2,800 s/mm^2^, directions = 140, TR: 7970 ms, TE: 102 ms, Flip Angle: 90^°^, FOV: 232 mm, acquisition matrix: 116 × 116, slice thickness: 2.0 mm, slices: 70.

The post-treatment protocol included an axial 3D IR-FFE T1-WI (TI: 450 ms, TR: 7.9 ms, TE:2.6 ms, Flip Angle: 8^o^, FOV: 240 mm, acquisition matrix: 240 × 240, slice thickness: 1.0 mm, slices: 170).

### 2.4. Segmentation of ablation site

**Figure 1:**
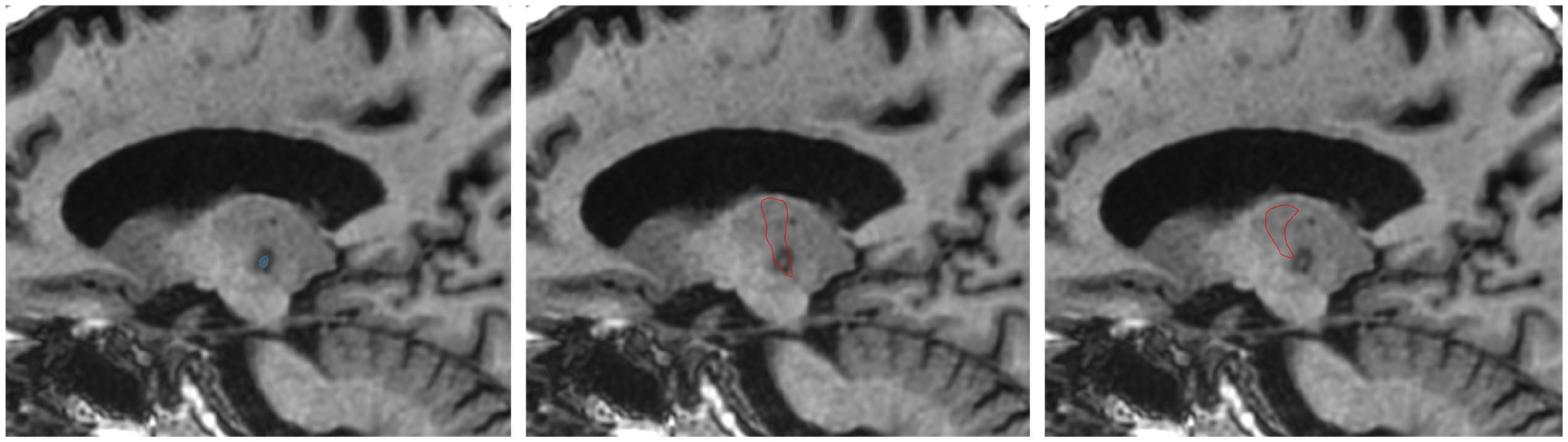
Example of ablation core segmented on T1-WI acquired 1 day post-treatment (left). FreeSurfer segmented VLp overlayed on day 1 T1-WI (middle). THOMAS VLp overlayed on day 1 T1-WI (right).

The FUS ablation site was identified and demarcated on the post-treatment T1-WI. The post-treatment T1-WI was first linearly co-registered to the pre-treatment T1-WI with FSL-FLIRT (23). To avoid including vasogenic and cytotoxic oedema, only the T1 iso-intense lesion core was included in the segmentation (Figure 1), corresponding to ablation zone 1 (24). The segmentation was performed with ITK-SNAP (25) by a trained neuroimaging analyst. While zone 2 can also form part of the final lesion necrotic core and is commonly included in ablation segmentations, zone 1 was isolated to improve the specificity of ablation overlap measurements, and reduce the risk of artificially inflating overlapping volumes with regions that may not form part of the necrotic core.

### 2.5. Thalamic parcellation

Two automated methods for segmentation of the thalamus into individual nuclei were applied to the pre-treatment T1-WI: THOMAS and FreeSurfer. The FreeSurfer thalamic segmentation module segments the thalamus into 26 thalamic nuclei, while THOMAS segments the thalamus into 11 nuclei. Both atlases provide a segmentation of the VLp, as per the Morel nomenclature (26), which includes the supposed location of the Vim as labelled by Hassler (27). Both thalamic segmentation techniques result in a hard segmentation, whereby each voxel in the segmented volume can belong to only one category, i.e. unique thalamic nuclei.

### 2.6. Brain tissue volumes

The T1-WI was n4 bias corrected and skull-stripped using an in-house AI-based brain extraction algorithm, followed by manually correction when necessary. A skull mask was generated using FSL-BET (28), and the images were processed with FSL-SIENAX (29) to derive estimates of absolute and skull size normalised whole brain, and substructure volumes.

The brain extracted T1-WIs from all subjects were combined to generate an unbiased template T1-WI using buildtemplateparallel in ANTS (30). Region of interest (ROI) masks were generated on the pre-treatment T1-WI for use in diffusion MRI tractography. Masks of the thalamus and precentral gyrus were extracted directly from the FreeSurfer segmentation and transformed to the native DWI space. Masks of the dentate nucleus and red nucleus were defined manually on the template T1-WI and transformed to the native T1-WI *via* non-linear registration using the inverse warp generated during the T1-WI template construction described above.

### 2.7. Diffusion MRI processing

The diffusion MRI data was processed using the default MRtrix3 (31) pipeline. Briefly, the raw DWI data were denoised, corrected for Gibb’s ringing, susceptibility distortion, eddy current corrected and bias field corrected. Fibre orientation distributions (FOD) (32) were estimated using single-shell multi-tissue spherical deconvolution (33) for the single shell data (protocol 1), and multi-shell multi-tissue spherical deconvolution (34) for the multi-shell data (protocols 2 and 3). Probabilistic fibre tracking (35) was used to reconstruct the DRTT, seeding in the dentate nucleus, with inclusion masks in the contralateral red nucleus, thalamus and pre-central cortex (36). Fibre tracking was terminated after 3,000 streamlines satisfied the inclusion criteria, and then reduced to the 1,000 most coherent streamlines with DIPY (37).

### 2.8. DRTT cluster segmentation

Due to the large size of the tractography inclusion masks, the path of the resultant streamlines through the thalamus exhibited several common paths, or clusters (Supplementary Figure 1). To determine which of these clusters was the most clinically relevant target, we created unbiased template clusters, which were warped and analysed in the individual subject space.

The white matter FOD of each subject was first processed to generate a total apparent fibre density (AFD) map, which gives the total density of all fibre populations within each voxel (38). The AFD images were combined to generate an unbiased template image using the command buildtemplateparallel in ANTS. Ten subjects were selected at random, and the native DRTT streamlines were warped to the AFD template. For each of the 10 subjects, the DRTT in the template space was split into three clusters—anterior (aDRTT), middle (mDRTT) and posterior (pDRTT), based on the trajectory of the streamlines between the red nucleus and thalamus using QuickBundles (39) in DIPY, an automated algorithm that groups streamlines together based on the similarity of location in space of each point along the entire streamline. Anterior, middle and posterior clusters were chosen due to the simplicity of implementation in a systematic fashion across multiple subjects. Adjustments to the threshold value in QuickBundles can be used to increase/decrease the number of clusters in the data. For each subject, the threshold value was incrementally increased until the resultant clusters included a posterior, middle and anterior component, as determined by manual inspection of the path of the cluster centroids. The selected cluster centroids were isolated, and each streamline in the full warped streamline set was assigned to one of the three clusters based on the path similarity between each streamline and the cluster centroid. While more than 3 clusters may have been present in the clustered data, streamlines were assigned to only one of the 3 selected clusters, corresponding to posterior, middle and anterior clusters (Supplementary Figure 1).

For each cluster, the streamlines from each subject were combined and filtered down to the 1,000 most coherent streamlines for that cluster, resulting in 3 template streamline bundles. Each bundle was warped back to the native DWI space of each subject. This process was performed independently for the left and right DRTT.

### 2.9. Ablation overlap

**Figure 2:**
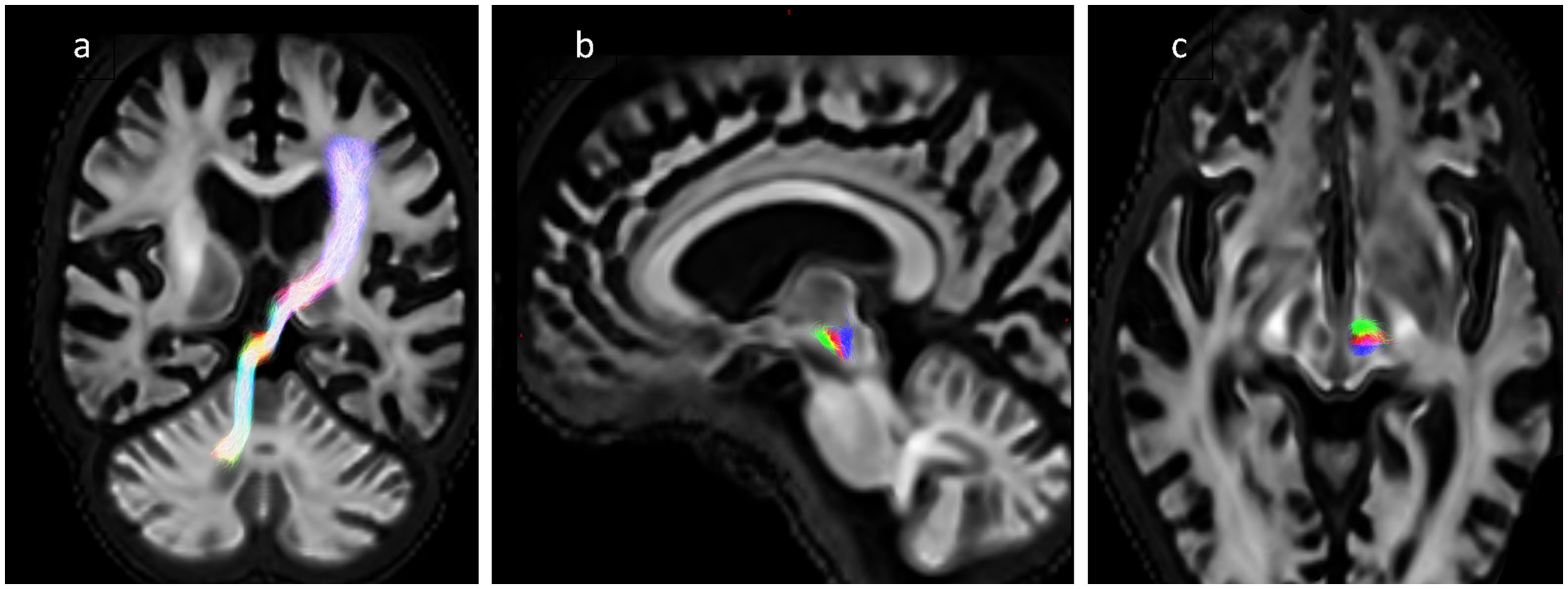
**(A)** Obliqued coronal view of native DRTT trajectory. **(B,C)** Sagittal and axial view of 3 template DRTT clusters in a single slice—anterior (green), middle (red) and posterior (blue).

The overlap between the ablation segmentation and the tractography was assessed by counting the number of streamlines that pass through the segmentation and dividing by the total number of streamlines in the bundle, where an overlap of 100% represents complete transection of the fibre tract. For each subject, there were 4 tracts: the DRTT generated from the native DWI data, and the 3 template bundles described above (aDRTT, mDRTT and pDRTT). An example of the native DRTT and three template clusters is shown in Figure 2.

The overlap of the ablation segmentation with each of the thalamic nuclei generated by FreeSurfer and THOMAS was calculated by multiplying each nuclei segmentation with the ablation segmentation mask and calculating the volume of the resulting overlap mask. The ablation overlap was calculated individually with all regions defined by the two atlases, to determine if the ablation overlap with any region was predictive of clinical outcome.

### 2.10. MRgFUS treatment parameters

Patient skull characteristics, such as skull density ratio (SDR), are often communicated with a single value; however, these values are not necessarily consistent across the entire skull. Measurements are taken along the beam from each of the 1,024 elements in the ultrasound array, and the mean value is typically used clinically. Given the array of values, it is possible to calculate additional summary statistics, such as the standard deviation. For each subject, the MRgFUS treatment parameters were extracted from the ExAblate console. The patient-specific parameters included skull density ratio (SDR), skull thickness (ST) and inner and outer skull angle. For each parameter, a value was extracted for each of the 1,024 elements in the ultrasonic array, and both the mean and standard deviation for each parameter were calculated for use in subsequent statistical analysis.

Treatment-specific parameters including the maximum temperature reached during the procedure, the number of active elements of the first sonication, the number of total sonications and the sum of temperature delivered across all sonications were also extracted.

### 2.11. Clinical assessment

Clinical evaluation of each patient was performed by a neurologist prior to treatment and again at regular intervals up to 36 months following treatment. The mean (±SD) length of the clinical follow-up visit from the treatment date was 16.0 ± 10.9 months. The evaluation included the clinical rating scale for tremor (CRST) (40) and hand tremor score (HTS) (7). As not all subjects returned for all follow-up evaluations, the latest available CRST and HTS score for each subject was used to assess tremor change following treatment. CRST is divided into three parts: A (CRST_A_), B (CRST_B_), and C (CRST_C_). CRST_A_ evaluates tremor by body region for the whole body, CRST_B_ assesses task performance and CRST_C_ assesses tremor related disability. The maximum possible score for the combined CRST (CRST_T_) is 160. The HTS is derived from CRST and comprises upper limb items including rest, postural, action tremor, pouring test, handwriting, constrained spirals (large and small) and constrained straight lines, each item scored 0–4. Because handwriting is only tested in the dominant hand, the HTS is out of 32 in the dominant hand and 28 in the non-dominant hand. As the absolute change in tremor scores can be expected to be greater in subjects with greater pre-treatment scores, percentage change in each tremor score was calculated such that a positive value indicated a reduction in tremor score.

### 2.12. Statistical analysis

Predictors of tremor suppression were assessed *via* multivariate linear regression by forward selection. Each predictor was added to the model individually, and the predictor with the lowest *p*-value under 0.05 was chosen to be included in the model. This process continued iteratively until there were no remaining predictors with a *p*-value < 0.05. An initial model was constructed including only patient-specific pre-treatment variables including age, sex, brain tissue volumes, SDR, skull thickness and inner and outer skull angle and pre-treatment tremor scores. Treatment-specific parameters including ablation volumes and overlap with the DRTT and thalamic nuclei segmentations, maximum temperature reached during the procedure, the number of active elements, the number of total sonications and the total sum of temperature delivered were then individually added to the model and again chose by forward selection. The goodness of fit of this more complex model was then compared to the baseline model *via* ANOVA comparison of residual sum of squares.

This process was repeated using all subcomponents of CRST (CRST_A_, CRST_B_, and CRST_C_), CRST_T_ and the dominant and non-dominant hand HTS values (HTS_D_ and HTS_ND_) as the dependent variable.

To remove any effect of secondary lesions on the observed statistical relationships, the statistical analysis was repeated for any statistically significant predictors, after exclusion of the 9 subjects who were lesioned in a secondary region outside the Vim.

All statistical analyses were performed using the R (version 4.2.2) statistical software package with RStudio (Version 1.4.1717, RStudio, Inc., Boston, MA URL, http://www.rstudio.com).

## 3. Results

### 3.1. Clinical outcomes

Clinical scores are summarised in Table 2. The mean reduction in CRST_T_ at the most recent follow-up visit was 44.77% ± 16.6%, while the mean reduction in HTS_D_ and HTS_ND_ was 62.58% ± 18.77% and 3.48% ± 23.90%, respectively. Both CRST_T_ and HTS_D_ were reduced in all 31 participants, while HTS_ND_ was reduced in 17 subjects.

**Table 2:** Mean change in tremor scores between pre-treatment and the most recent clinical visit following treatment with MRgFUS.

Variable	Pre-treatment	Post-treatment	Tremor change (%)
**CRST**			
Part A	23.1	13.16	41.69
Part B	21.48	13.45	38.6
Part C	14.68	6.67	56.31
Total	60.03	33.68	44.77
**HTS**			
Dominant hand	19.03	7.48	62.58
Non-dominant hand	16.19	15.52	3.48

### 3.2. Ablation volumes

The mean ablation core volume at 1 day post-treatment was 11.61 ± 4.82 mm^3^. The FreeSurfer defined nuclei with the greatest overlapping ablation volume was the ventral lateral posterior (VLp) nucleus 5.13 ± 3.64 mm^3^ followed by the ventral lateral anterior (VLa) 2.30 ± 2.02 mm^3^. Similarly, the VLp defined by THOMAS showed the greatest overlap with the ablation (2.51 ± 2.90 mm^3^).

**Figure 3:**
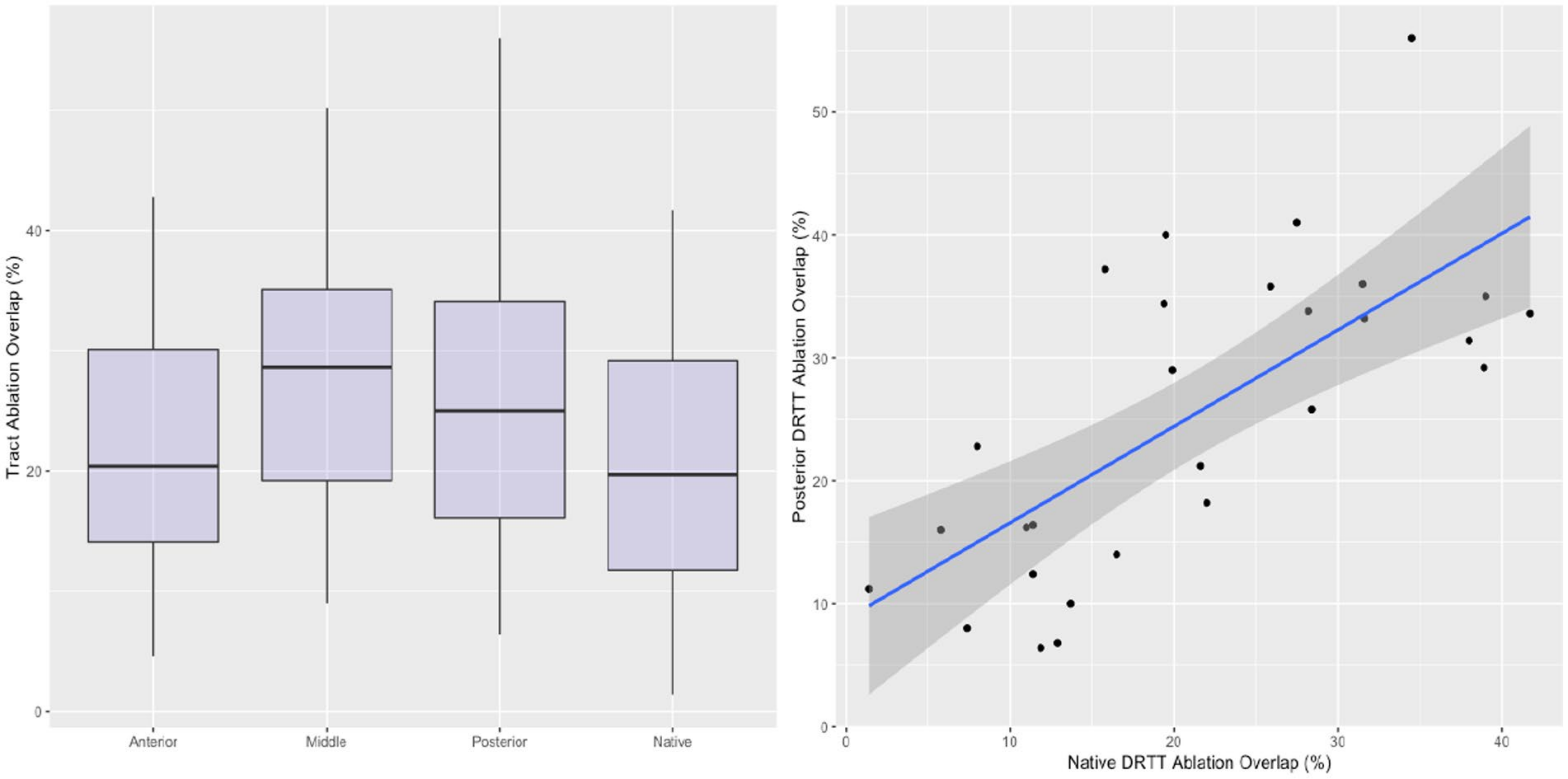
Boxplots displaying the ablation overlap for the native DRTT and 3 template DRTT clusters (left). Correlation between the ablation overlap between the native DRTT and the posterior template DRTT (right). Line of best fit indicated by blue line, and 95% confidence interval indicated by shaded region.

The percentage of streamlines overlapping with the ablated volume are shown in Figure 3. Overlap with the ablation was greatest in the mDRTT (28.35% ± 11.66%), followed by the pDRTT (25.28% ± 12.14%), and aDRTT (21.79% ± 10.56%) clusters, and the native DRTT (20.86% ± 11.33%). The ablation overlap with the native DRTT was significantly correlated with both the mDRTT and pDRTT clusters (r = 0.445 and 0.473, respectively), per Pearson correlation analysis; however, the correlation was stronger with the posterior cluster (R^2^ = 0.224) than the middle cluster (R^2^ = 0.198), indicating that the path of the native DRTT through the thalamus more closely followed that of the pDRTT. Pearson correlation between the ablation overlap of the native DRTT and aDRTT was not statistically significant (r = 0.263, *p* = 0.105).

### 3.3. Predictors of tremor suppression

**Figure 4:**
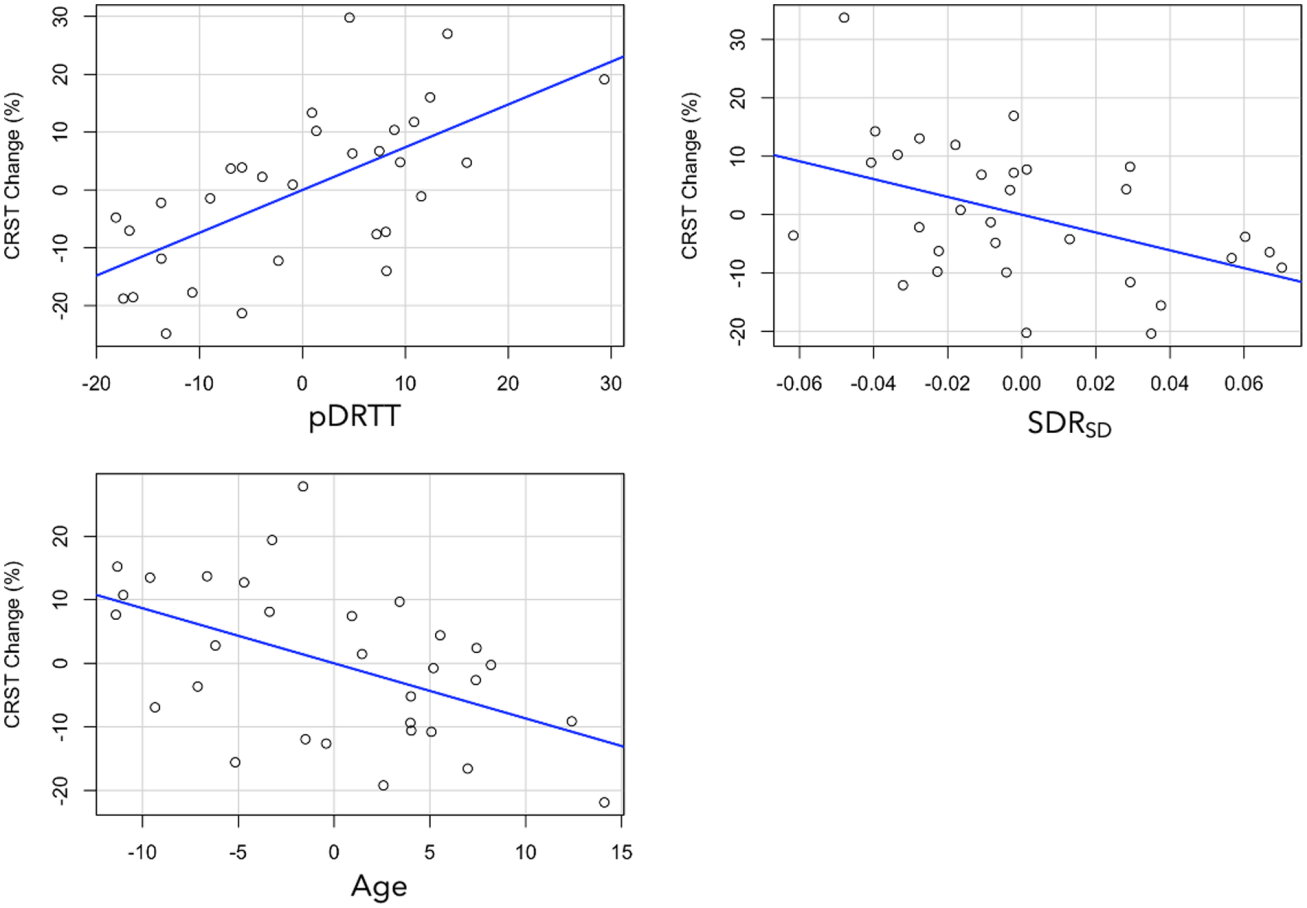
Partial plots from multivariate regression with CRST change as dependent variable for pDRTT (top left), SDR_SD_ (top right) and age (bottom left). All variables are mean centred.

Multivariate linear regression, with percentage CRST_T_ change as the dependent variable revealed that of the patient-specific parameters, age (β = −0.375, *p* = 0.006) and SDR standard deviation (SDR_SD_; β = −0.324, *p* = 0.015) were statistically significant predictors. After addition of treatment-specific parameters to the model, total ablation core volume (β = 0.318, *p* = 0.023) and pDRTT overlap (β = 0.533, *p* < 0.001) were also statistically significant predictors (Figure 4); however, the model including pDRTT as a predictor (R^2^ = 0.548) provided a significantly better model fit, per ANOVA model comparison (*p* < 0.001), while the model including the total ablation core volume (R^2^ = 0.408) did not provide a significant model improvement (*p* = 0.08).

Replacement of CRST_T_ with each of the CRST subcomponents revealed that pDRTT overlap was a significant predictor of CRST_A_ (β = 0.452, *p* = 0.007) and CRST_B_ (β = 0.377, *p* = 0.040) change, while it was not a significant predictor of change in CRST_C_. SDR_SD_ was approaching significance for CRST_A_ change (β = −0.319, *p* = 0.052), but was not a significant predictor of change in CRST_B_ or CRST_C_. Patient age was not a significant predictor of change in any CRST subcomponent.

Change in HTS_D_ was found to be significantly negatively associated with age (β = −0.576, *p* < 0.001), while the relationship with SDR_SD_ was approaching statistical significance (β = −0.256, *p* < 0.081). HTS_ND_ change was found to be significantly associated with pDRTT overlap (β = 0.472, *p* = 0.006). No treatment-specific metrics, brain tissue volumes or pre-treatment tremor scores were found to be significant predictors of percentage change in CRST or HTS.

When the analysis was repeated excluding the nine patients with secondary lesions, pDRTT overlap remained a significant predictor of change in CRST_A_ (β = 0.588, *p* = 0.006), CRST_T_ (β = 0.562, *p* = 0.003) and HTS_ND_ (β = 0.522, *p* = 0.016). SDR_SD_ was a significant predictor of change in CRST_A_ (β = −0.401, *p* = 0.038) and CRST_T_ (β = −0.383, *p* = 0.022), and approaching significance for HTS_D_ change (β = −0.365, *p* = 0.056).

Ablation overlap volumes with the THOMAS and FreeSurfer derived thalamic segmentations were not found to be significant predictors of CRST or HTS change.

## 4. Discussion

In this study, we sought to investigate the relationship between the change in tremor scores following treatment with MRgFUS, and the location of the ablated volume in relation to a number of potential targeting methods. Additionally, we investigated the relationship between several patient-specific characteristics and tremor change, to determine if these characteristics were predictive of clinical outcome.

We observed a significant relationship between the degree of DRTT lesioning and tremor suppression at follow-up, following treatment with MRgFUS. Our results suggest that while the middle DRTT cluster is the region most consistently lesioned across all subjects, it is the degree of posterior DRTT lesioning that has the greatest impact on tremor suppression. These results do not necessarily suggest that surgeons should forgo targeting of the middle DRTT in favour of the posterior region, rather, it is perhaps the extension of the lesion into the posterior fibres that has resulted in the additional clinical benefit observed in this patient cohort, consistent with the findings of Pineda-Pardo et al. (41) and Boutet et al. (42) who found that clinical improvement was corelated with more posteriorly placed lesions.

The presence of the non-decussating DRTT (nd-DRTT) might explain the importance of the posterior region of the DRTT in achieving optimal clinical outcomes. The nd-DRTT consists of fibres that do not decussate from the dentate nucleus to the contralateral red nucleus and thalamus, representing around 20% of the total DRTT fibres (43). The course of the nd-DRTT is medial and posterior to the d-DRTT (15), and it has been postulated that the Vim is located at the point of anterior–posterior fading between these two tracts (44). Thus, the results presented here could indicate that extension of the ablation into the posterior region of the DRTT, towards the region typically occupied by fibres of the nd-DRTT could result in improved clinical outcomes. While this hypothesis is supported by our finding that tremor in the non-dominant hand was significantly associated with pDRTT lesioning, while tremor in the dominant hand was not, further studies specifically designed to investigate these findings are warranted.

The methodology presented in this work for isolation of the posterior fibres of the DRTT has the potential to provide a more specific fibre tract than the conventionally generated DRTT. Such methods require extensive validation before they can be adopted for treatment targeting, however the results presented here suggest further investigation into this method is warranted. We believe that while the native DRTT is not incorrect, the probabilistic tractography algorithm often required to generate the d-DRTT can result in additional unrealistic spurious streamlines that need to be pruned to reveal the true course of the DRTT. By defining this course in a template space and then warping the streamlines to individual subject space, we alleviate the impact of non-realistic streamlines, and through use of the AFD image to generate the non-linear warp field, we retain the intra-thalamic contrast necessary to accurately define the region of interest.

Although not as significant as pDRTT overlap, we observed that the total lesion core volume was also a significant predictor of percentage CRST reduction; however, this relationship is likely owing to the strong correlation between the total lesion core volume and the pDRRT overlap (r = 0.643, *p* < 0.001), which is supported by our finding that inclusion of the total lesion volume did not significantly improve the model for CRST change. Lesion volume in the FreeSurfer and THOMAS-derived VLp segmentation did not correlate with improvement in any tremor score. This result may be a consequence of the fact that both methods segment the entire VLp, rather than the Vim specifically, resulting in a less specific target volume.

In addition to the location of the MRgFUS lesion, the effectiveness of treatment is also limited by the ability to achieve sufficient tissue destruction to create long-lasting microstructural change. A number of patient characteristics such as SDR (45–51) and skull angle (48) have previously been shown to be important factors in the delivery of thermal energy to the treatment target. It is well documented that lower SDR results in less efficient energy transmission across the skull (45–52), requiring greater sonication power to achieve the same temperature elevation. However, numerous studies have demonstrated that successful tremor suppression can still be achieved at low SDR, by delivery of longer duration, low temperate doses (45, 47, 53–55). The results of this investigation suggest that it may be the variance in SDR values across the skull, rather than the mean value, that is more relevant for achieving clinically effective lesions, and thus optimal outcomes. While exploration of the relationship between SDR uniformity and lesion formation was beyond the scope of this study, this finding could help explain the observed heterogeneity in outcomes, and should be studied further.

Our finding that older age was negatively associated with sustained tremor reduction is consistent with previous findings (56) and highlights the importance of patient age as well as SDR, in the screening of appropriate patients for treatment of tremor with MRgFUS.

This study had several limitations. The variation in DWI protocol across subjects undoubtedly contributed additional variance to the DRTT measurements; however, ANOVA analysis of the results between the 3 protocols revealed that there was not a systematic difference in results observed from the different protocols. Additionally, a lack of follow-up imaging data limited the image analysis to cross-sectional, preventing analysis of microstructure change in the DRTT. The inclusion of subjects treated in additional regions outside of the Vim may have influenced the results; however, repetition of the statistical analysis with these subjects excluded did not change the outcomes of this investigation.

## 5. Conclusion

In this investigation, we report that CRST improvement following lesioning of the Vim with MRgFUS was predicted by patient age, SDR standard deviation and the degree of the lesioning in the posterior DRTT. Lesion volume in the FreeSurfer and THOMAS derived VLp was not associated with change in tremor scores. These results may suggest that tractography-based methods could provide more specific treatment target compared with atlas based methods; however, further validation is required before such techniques can be adopted clinically.

## Data availability statement

The raw data supporting the conclusions of this article will be made available by the authors, without undue reservation.

## Ethics statement

The studies involving human participants were reviewed and approved by St Vincent’s Hospital Ethics Review Committee (ETH00670). The patients/participants provided their written informed consent to participate in this study.

## Author contributions

KK contributed to the study conception, data analysis and interpretation, and drafting the article. JMal, MB, and AD’S contributed to the study conception and critical revision of the article. YB and BJ contributed to the data collection and critical revision of the article. FC, JMaa, and JP contributed the critical revision of the article. CW contributed to the study conception, critical revision of the article and approval of the version to be published. ST contributed to the study conception, data collection, critical revision of the article and approval of the version to be published. All authors contributed to the article and approved the submitted version.

## Funding

KK was supported by GE Healthcare Australia Medical Physics PhD Scholarship.

## Conflict of interest

KK declares that he was the recipient of funding in the form of a PhD scholarship from GE Healthcare Australia. MB reports research support from Biogen, Novartis, Sanofi-Genzyme, Merck, Alexion and Bristol Myers Squibb and advisory board consulting fees from Novartis and Autobahn Therapeutics. MB is the Director of Research (Honorary), Sydney Neuroimaging Analysis Centre, and *ad-hoc* consultant, RxMx.

The remaining authors declare that the research was conducted in the absence of any commercial or financial relationships that could be construed as a potential conflict of interest.

## Publisher’s note

All claims expressed in this article are solely those of the authors and do not necessarily represent those of their affiliated organizations, or those of the publisher, the editors and the reviewers. Any product that may be evaluated in this article, or claim that may be made by its manufacturer, is not guaranteed or endorsed by the publisher.
