# Supplementary material for: Tremor suppression following treatment with MRgFUS: skull density ratio consistency and degree of posterior dentatorubrothalamic tract lesioning predicts long-term clinical outcomes in essential tremor
Source: Front Neurol. 2023 Apr 25;14:1129430. doi: 10.3389/fneur.2023.1129430 (PMC10166854; doi:10.3389/fneur.2023.1129430)
Supplement: Supplementary file 1 [file Image_1.pdf]

Supplementary Material

DRTT Cluster Segmentation

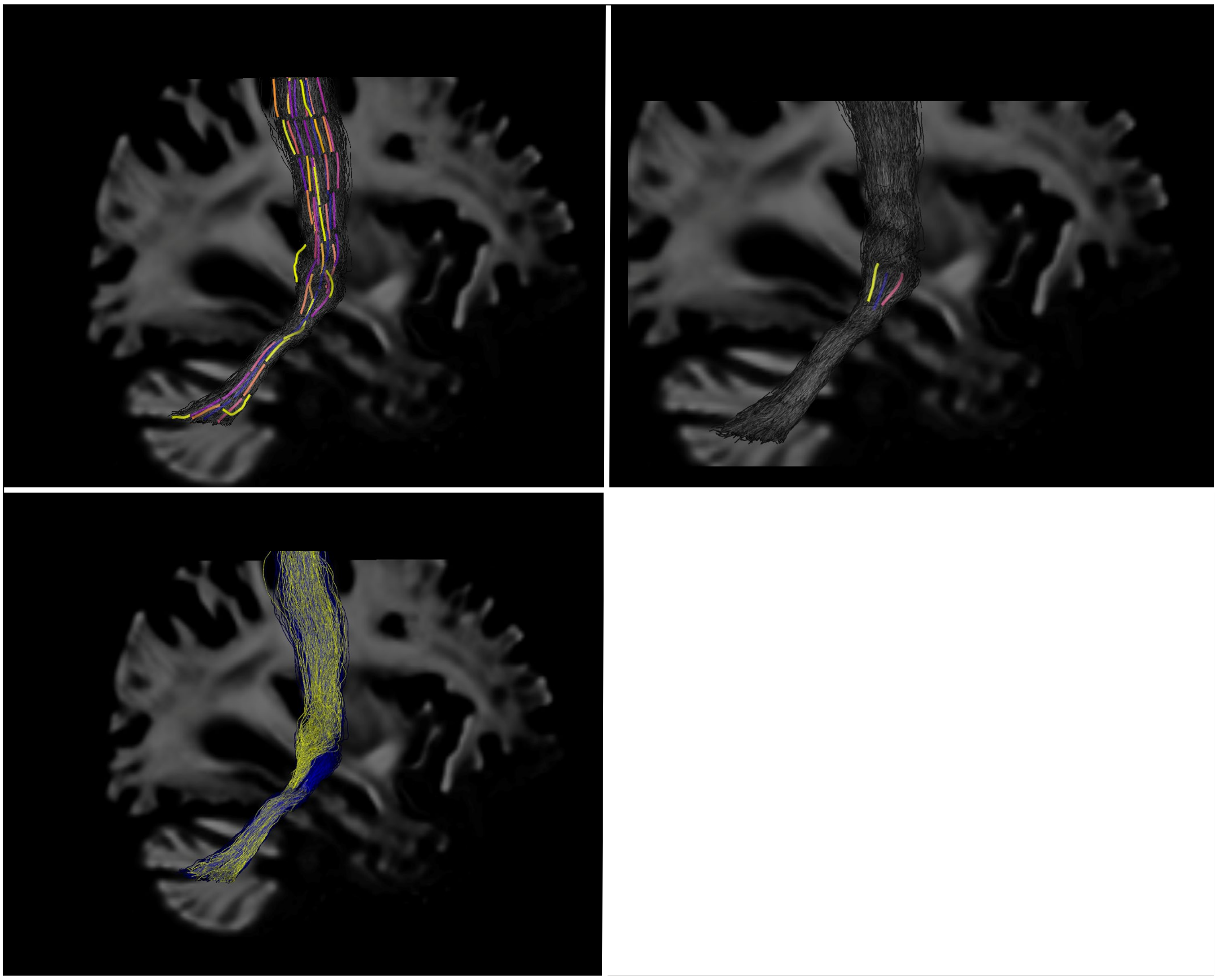

Figure 1. DRTT clustering process. **Top left**. For each subject, the DRTT streamlines in the unbiased AFD template space are broken up into 12 segments along the length of the tract. Each segment is further separated into clusters with QuickBundles[1] in DIPY, based on the trajectory of the streamlines within that segment only. Each cluster is represented with a coloured centroid that follows the average trajectory of streamlines in that cluster. **Top right**. Clusters in the segment between the red nucleus and thalamus were isolated, and the QuickBundles cluster threshold was adjusted until the clusters included a posterior (yellow), middle (blue) and anterior bundle (pink). **Bottom left.** Each streamline in the full warped streamline set is assigned to one of the three clusters based on the path similarity between each streamline and the cluster centroid. In this example the streamlines assigned to posterior cluster are shown in yellow, with the remaining streamlines in blue. This process was repeated individually for each of the 5 selected subjects, and the clustered streamlines combined to give the final DRTT clusters.

References

[1] E. Garyfallidis, “Towards an Accurate Brain,” no. May, pp. 1–181, 2012.
